# Optimization Algorithm for Kalman Filter Exploiting the Numerical Characteristics of SINS/GPS Integrated Navigation Systems

**DOI:** 10.3390/s151128402

**Published:** 2015-11-11

**Authors:** Shaoxing Hu, Shike Xu, Duhu Wang, Aiwu Zhang

**Affiliations:** 1 School of Mechanical Engineering and Automation, Beihang University, Beijing 100191, China; E-Mails: xushike24k@163.com (S.X.); buaaben@163.com (D.W.); 2 Ministry of Education Key Laboratory of 3D Information Acquisition and Application, Capital Normal University, Beijing100089, China; E-Mail: zhang_aiwu@126.com

**Keywords:** computational optimization, SINS/GPS, closed-loop Kalman filter, block matrix, offline-derivation, parallel processing, accuracy-lossless decoupling, symbol operation

## Abstract

Aiming at addressing the problem of high computational cost of the traditional Kalman filter in SINS/GPS, a practical optimization algorithm with offline-derivation and parallel processing methods based on the numerical characteristics of the system is presented in this paper. The algorithm exploits the sparseness and/or symmetry of matrices to simplify the computational procedure. Thus plenty of invalid operations can be avoided by offline derivation using a block matrix technique. For enhanced efficiency, a new parallel computational mechanism is established by subdividing and restructuring calculation processes after analyzing the extracted “useful” data. As a result, the algorithm saves about 90% of the CPU processing time and 66% of the memory usage needed in a classical Kalman filter. Meanwhile, the method as a numerical approach needs no precise-loss transformation/approximation of system modules and the accuracy suffers little in comparison with the filter before computational optimization. Furthermore, since no complicated matrix theories are needed, the algorithm can be easily transplanted into other modified filters as a secondary optimization method to achieve further efficiency.

## 1. Introduction

In many SINS/GPS integrated navigation systems, common system models cannot conform to the use conditions of classical Kalman filter due to the “colored noise”. To still be able to use the optimal estimation method, it is necessary to expand the system state model [1,2]. However, as the state dimensions increase, filtering computation will rapidly become expensive and unstable, and the so-called “dimension disaster” could even break out. In order to address this problem, scholars have reported various optimization algorithms which can lower the computational costs and/or enhance the numerical robustness. The typical optimization algorithms contain square-root information filtering SRIF [3], U-D decomposition filtering [4], singular value decomposition filtering [5] and their improved versions [6,7,8]. These modified filters, besides greatly reducing the time consumption, theoretically guarantee the positive definiteness of covariance and effectively avoid the numerical divergence, thus they are widely applied in the theoretical design of higher-order systems. Nevertheless, they require the support of relatively complex matrix theory in derivation and may pose certain difficulty in engineering. On the other hand, although these algorithms have decreased computational costs, their complexity is still *O*(*n*^3^). With further expansion of the state dimensions, the above decomposition algorithms, as a kind of general method, are also being challenged on their real-time capability. Recently, researchers have paid much attention to non-commonality methods, which are designed for some specific applications or based on specific models. For instance, aiming at integrated navigation applications, a kind of reduced-order Kalman filter (RDKF) [9,10,11,12] was promoted to ease the computational load. The idea of these filters is to reduce the model dimension by theoretical/engineering methods. As the filter dimension *n* is vital to computation time, the reduction of the state order will produce a direct benefit in terms of real time. However, the decrease of the state order will also bring partial accuracy damage, therefore, these methods may not be suitable for some high accuracy-demanding applications. Another kind of effective algorithm optimizes the float-point operations mainly by taking advantage of the sparse matrices (e.g., the transition matrix *Φ_n_* in SINS/GPS) during the filtering computation [13,14,15]. In these algorithms, the so-called matrix accumulative method (MAM) or other online methods are used and the algorithm complexity can be simplified to *O*(*s^2^ – u^2^*) (*s/u* represents the numbers of nonzero/1 elements in *Φ_n_*). As the matrices in the high order model normally contain substantial numbers of zero elements, the computational time can be curtailed to an ideal level by usage of this sparse-matrix-based method. Meanwhile, being different from RDKF, the sparse-matrix-based methods do not change the system model and need little engineering approximation, thus they perform better in the accuracy-control aspect. But these methods also have their own limitations:

(1) Though they avoid massive unnecessary multiplying-zero operations, the methods still need extra *O*(*n*^3^) times estimation of zero elements in each matrix multiplication;
(2) Methods based on MAM, a kind of online method, could not seek any deeper optimization online and their efficiency is totally dependent on the number of zeros in the real-time matrix. To enhance the optimization efficiency, the usual way is to set more zero elements by approximation methods, but this comes at the cost of a certain accuracy loss.

In response to the above problems, we present here a new highly-efficient, accuracy-lossless, and engineering-tractable optimization algorithm based on offline derivation and a parallel method exploiting the numerical characteristics of SINS/GPS. In comparison with other algorithms, the proposed method offers numerous advantages: (i) in comparison with the general algorithms, our proposed method requires little complex deduction and is easily understandable; (ii) in comparison with the RDKF optimization method, the proposed method needs little engineering approximation and would be more accurate; and (iii) in comparison with the filter based on MAM, it is free of zero-estimation operations and can deduce some stronger conclusions, thus it is more efficient. Emphasizing on the engineering simplicity, we will introduce our optimization method revolving around the classical Kalman filter and a loosely-coupled model of SINS/GPS. It is apprised here that the chosen model does not imply any constraints on the application scope of our method. Actually, the offline and parallel method, as a pure numerical optimization method, is equally applicable to the extended filter and other complex SINS/GPS models if some similar research on the special model is done. On the other hand, since there is no transformation on any models or equations in our method (the two distinct differences with the normal filtering computations are: (i) unnecessary floating-point operations are directly avoided; and (ii) de-coupled operations are processed in parallel), the precision and robustness of our method would be equal to the classical Kalman filter in theory. Owing to this fact, the efficiency rather than robustness or accuracy is focused on in our discussion later. Section 2 gives a brief account of closed-loop Kalman filtering and a widely-used loosely-coupled model in SINS/GPS. In Section 3, the details of offline derivation and the parallel method based on a block-matrix technique are described. Section 4 and Section 5 deal with the statistics on computational costs and the filter performance on error estimation.

## 2. Loosely-Coupled SINS/GPS Model Using the Classical Kalman Filter 

Depending on the different ways of integration, SINS/GPS are classified into two modes: (i) loose mode; and (ii) tight mode. Loose mode using GPS output to adjust SINS errors is opted for in this research for its distinctive features of high redundancy and easy realization. The emphasis is placed on a common kind of loose model *i.e.*, position-velocity integration with 18 states and six observations.

### 2.1. State Equation

By using the indirect method, which treats the estimation variables as parametric errors instead of parameters themselves, the state equation is established in Equation (1):

(1) $$\dot{x}\left(t\right)=A\left(t\right)x\left(t\right)+G\left(t\right)\omega \left(t\right)$$

where, x(t) represents the state vector, including attitude errors γ, velocity errors δv, position errors ${\left(\begin{array}{ccc} \delta L\; & \delta \lambda & \delta h \end{array}\right)}^{\text{T}}$, and the “colored noise” errors: gyro constant drift $\varepsilon_{c}$, Markov process drift $\varepsilon_{r}$, and accelerometer drift $\nabla_{a}$; ω(t) denotes the collected system noise vector, including gyro drift white noise $\omega_{g}$, Markov driving white noise $\omega_{r}$, and accelerometer drift white noise $\omega_{a}$; A(t) is state transition matrix and G(t) is noise coefficient matrix. Combining references [16,17,18,19,20,21,22] with some modification, the variables of the state equation can be described by Equations (2)–(5):

(2) $$x(t)={\left(\gamma_{x}\;\gamma_{y}\;\gamma_{z}\vdots \delta v_{x}\;\delta v_{y}\;\delta v_{z}\vdots \delta L\;\delta \lambda \;\delta h\vdots \varepsilon_{cx}\;\varepsilon_{cy}\;\varepsilon_{cz}\vdots \varepsilon_{rx}\;\varepsilon_{ry}\;\varepsilon_{rz}\vdots \nabla_{ax}\;\nabla_{ay}\;\nabla_{az}\right)}^{\text{T}}$$

(3) $$\omega \left(t\right)={\left(\omega_{gx}\;\omega_{gy}\;\omega_{gz}\vdots \omega_{rx}\;\omega_{ry}\;\omega_{rz}\vdots \omega_{ax}\;\omega_{ay}\;\omega_{az}\right)}^{\text{T}}$$

(4) $$A(t)={\left[\begin{array}{cccccc} A_{11} & A_{12} & A_{13} & C_{B}^{N} & C_{B}^{N} & O_{3\times 3}\\ A_{21} & A_{22} & A_{23} & O_{3\times 3} & O_{3\times 3} & C_{B}^{N}\\ O_{3\times 3} & A_{32} & A_{33} & O_{3\times 3} & O_{3\times 3} & O_{3\times 3}\\ O_{3\times 3} & O_{3\times 3} & O_{3\times 3} & O_{3\times 3} & O_{3\times 3} & O_{3\times 3}\\ O_{3\times 3} & O_{3\times 3} & O_{3\times 3} & O_{3\times 3} & A_{IMU22} & O_{3\times 3}\\ O_{3\times 3} & O_{3\times 3} & O_{3\times 3} & O_{3\times 3} & O_{3\times 3} & A_{IMU33} \end{array}\right]}_{18\times 18}$$

(5) $$G\left(t\right)={\left[\begin{array}{ccc} C_{B}^{N} & O_{3\times 3} & O_{3\times 3}\\ O_{3\times 3} & O_{3\times 3} & O_{3\times 3}\\ O_{3\times 3} & O_{3\times 3} & O_{3\times 3}\\ O_{3\times 3} & O_{3\times 3} & O_{3\times 3}\\ O_{3\times 3} & I_{3\times 3} & O_{3\times 3}\\ O_{3\times 3} & O_{3\times 3} & I_{3\times 3} \end{array}\right]}_{18\times 9}$$

In matrices (4) and (5), all blocks are 3-order square matrices. Blocks $O_{3\times 3}$, $I_{3\times 3}$, and $C_{B}^{N}$ stand for the zero matrix, the identity matrix, and the attitude matrix, respectively. The other blocks are described in detail in [16,17,18,19,20,21,22]. In particular, we highlight here two kinds of nonzero blocks $A_{i3(i=1,2,3)}$ and $A_{IMUii(i=2,3)}$ of which the special single-nonzero-vector and diagonal structure may contribute to deeper optimization:

(6) $$A_{i3}=\left[\begin{array}{ccc} {}* & 0 & 0\\ {}* & 0 & 0\\ {}* & 0 & 0 \end{array}\right],\;i=1,2,3\;A_{IMUii}=\left[\begin{array}{ccc} {}* & 0 & 0\\ 0 & * & 0\\ 0 & 0 & * \end{array}\right],\;i=2,3$$

In addition, the variance Q(t) of noise vector ω(t) is described as:

(7) $$Q\left(t\right)={\left[\begin{array}{ccc} Q_{11} & O_{3\times 3} & O_{3\times 3}\\ O_{3\times 3} & Q_{22} & O_{3\times 3}\\ O_{3\times 3} & O_{3\times 3} & Q_{33} \end{array}\right]}_{9\times 9}$$

where, block $Q_{ii(i=1,2,3)}$ is also diagonal.

### 2.2. Measurement Equation

We adopt a position-velocity integration model and define the filter observation vector z(t) [16,22]:

(8) $$z_{obs}\left(t\right)=\left[\begin{array}{c} \left(L_{INS}-L_{GPS}\right)R_{n}\\ (\lambda_{INS}-\lambda_{GPS})R_{e}\cos L\\ h_{INS}-h_{GPS}\\ v_{xINS}-v_{xGPS}\\ v_{yINS}-v_{yGPS}\\ v_{zINS}-v_{zGPS} \end{array}\right]=\left[\begin{array}{c} R_{n}\delta L+N_{x}\\ R_{e}\cos L\delta \lambda +N_{y}\\ \delta h+N_{z}\\ \delta v_{x}+M_{x}\\ \delta v_{y}+M_{y}\\ \delta v_{z}+M_{z} \end{array}\right]$$

Then, we define the measurement equation as follows:

(9) z(t)≜H(t)x(t)+n(t)

where n(t) represents the measurement noise vector; R(t) denotes the corresponding variance matrix, and H(t) is the coefficient matrix. n(t), R(t) and H(t) are described as follows according to [16,17,18,19,20,21,22]:

(10) $$n\left(t\right)={\left(N_{x},N_{y},N_{z},M_{x},M_{y},M_{z}\right)}^{\text{T}}$$

(11) $$R\left(t\right)=\text{diag}\left\{\sigma_{px}^{2},\sigma_{py}^{2},\sigma_{pz}^{2},\sigma_{vx}^{2},\sigma_{vy}^{2},\sigma_{vz}^{2}\right\}=\left[\begin{array}{cc} R_{11} & O_{3\times 3}\\ O_{3\times 3} & R_{22} \end{array}\right]$$

(12) $$H\left(t\right)={\left[\begin{array}{cccccc} O_{3\times 3} & O_{3\times 3} & H_{13} & O_{3\times 3} & O_{3\times 3} & O_{3\times 3}\\ O_{3\times 3} & I_{3\times 3} & O_{3\times 3} & O_{3\times 3} & O_{3\times 3} & O_{3\times 3} \end{array}\right]}_{6\times 18}$$

where:

(13) $$H_{13}=diag\left\{R_{n}\;,R_{e}\cos L,1\right\}$$

### 2.3. Closed-Loop Kalman Filtering

In order to fulfill the requirement of closed-loop Kalman filtering, the state equation (Equation (1)) and measurement equation (Equation (9)) are changed to the discrete forms:

(14) $$x_{n}=\phi_{\;n}x_{n-1}+u_{cn}+\Gamma_{n}\omega_{n}$$

(15) $$z_{n}=H_{n}x_{n}+n_{n}$$

where, the transition matrix $\phi_{\;n}$ is converted from A(t); the noise driving matrix $\Gamma_{n}$ is converted from G(t); the coefficient $H_{n}$ is converted from H(t); and the control vector $u_{cn}={\left(\gamma_{c}^{T}\vdots \delta v_{c}^{T}\vdots \delta L_{c}\;\delta \lambda_{c}\;\delta h_{c}\vdots \varepsilon_{cc}^{T}\vdots \varepsilon_{rc}^{T}\;\vdots \nabla_{ac}^{T}\right)}_{c}^{T}$ is added for real-time calibration on the parameters of SINS.

After discretization, the update of $x_{n}$ is done recursively as follows:

(16) $$\begin{array}{l} {\tilde{x}}_{n}\left(-\right)=\phi_{\;n}{\tilde{x}}_{n-1}\left({+}_{c}\right)\\ {\tilde{z}}_{n}=H_{n}{\tilde{x}}_{n}\left(-\right)\\ {\tilde{x}}_{n}\left({+}_{e}\right)={\tilde{x}}_{n}\left(-\right)+K_{n}(z_{obsn}-{\tilde{z}}_{n})\\ {\tilde{x}}_{n}\left({+}_{c}\right)={\tilde{x}}_{n}\left({+}_{e}\right)+u_{cn} \end{array}$$

where, “~” represents the parameter needed estimation; (−) represents the value before estimation; $\left({+}_{e}\right)$ represents the value after estimation; $\left({+}_{c}\right)$ represents the value after calibration with the vector $u_{cn}$; $z_{obsn}$ represents real observation vector; and $K_{n}$ represents Kalman filtering gain.

To minimize the filtering error, state vector ${\tilde{x}}_{n}\left({+}_{c}\right)$ is permanently set to zero:

(17) $${\tilde{x}}_{n}\left({+}_{c}\right)\equiv 0$$

Substitution of Equation (17) into Equation (16), yields:

(18) $$u_{cn}=-{\tilde{x}}_{n}\left({+}_{e}\right)=-K_{n}z_{obsn}$$

**Figure 1:**
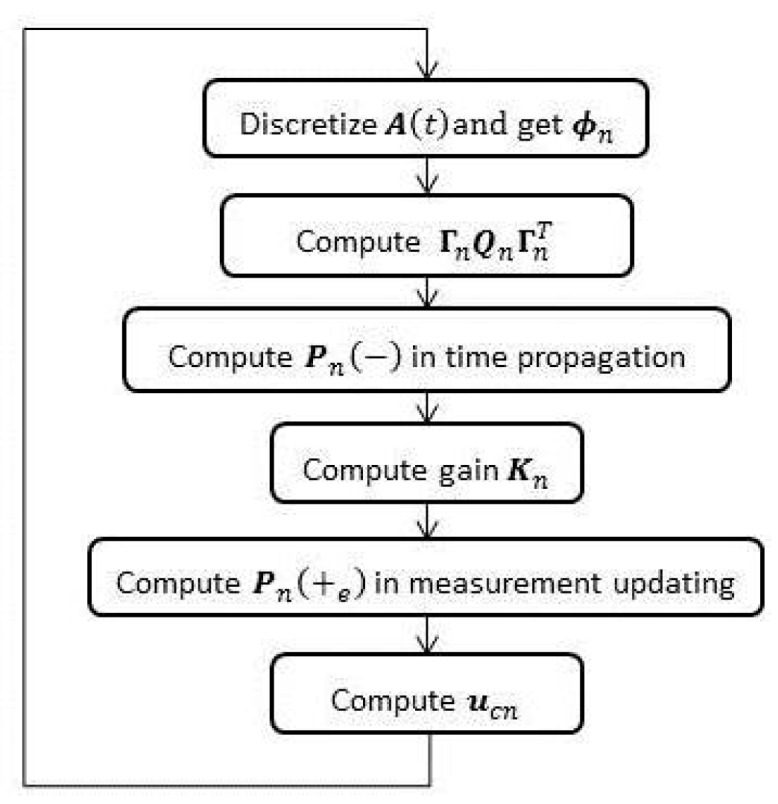
Iterative closed-loop Kalman filtering process.

Apparently, $u_{cn}$ becomes the final output of the Kalman filter with control vector. As $z_{obsn}$ is almost directly obtained by measurement, $K_{n}$ appears to be the key of $u_{cn}$ and even the filter. According to the Kalman filtering theory, $K_{n}$ can be recursively computed as follows:

(19) $$P_{n}\left(-\right)=\phi_{\;n}P_{n-1}\left({+}_{e}\right)\phi_{\;n}^{T}+\Gamma_{n}Q_{n}\Gamma_{n}^{T}$$

(20) $$K_{n}=P_{n}\left(-\right)H_{n}^{T}{\left(H_{n}P_{n}\left(-\right)H_{n}^{T}+R_{n}\right)}^{-1}$$

(21) $$P_{n}\left({+}_{e}\right)=\left(I-K_{n}H_{n}\right)P_{n}\left(-\right)$$

where $P_{n}$ is the covariance; $Q_{n}$ and $R_{n}$ are the discretized forms of Q(t) and R(t) respectively. In fact, Equations (18)–(21) constitute the main process of the closed-loop Kalman filter. This process is generally subdivided into two processes: time propagation Equation (19) and measurement updating Equations (18), (20) and (21). In summary, the process flow of Kalman filter in SINS/GPS is depicted in Figure 1. Since most matrices involved in Figure 1 or Equations (19)–(21) are either 18 × 18 or 18 × 6 matrices, there will be a large-scale and time-consuming computation in each recursive cycle. Therefore, a certain optimization on the computation would be essential to the real-time properties of SINS/GPS.

## 3. Computational Optimization

Consistent with Figure 1, optimization are sequentially implemented in the updating processes of $\phi_{\;n}$, $\Gamma_{n}Q_{n}\Gamma_{n}^{T}$, $P_{n}\left(-\right)$, $K_{n}$, $P_{n}\left({+}_{e}\right)$, where the most computational time are consumed.

### 3.1. Compute $\phi_{\;n}$

According to [16,17,18,19,20,21,22], we can calculate $\phi_{\;n}$ as follows:

(22) $$\phi_{\;n}=e^{\int_{t_{n-1}}^{t_{n}}A\left(t\right)dt}\approx I+T_{n}A_{n}+\frac{T_{n}^{2}}{2!}A_{n}^{2}+\frac{T_{n}^{3}}{3!}A_{n}^{3}$$

Here, the 3rd order Taloy equation is selected for mainly two reasons:

(1) Equation (22) meets the requirements of most mid-high-accuracy systems;

(2) Equation (22) can deduce the universal optimization conclusion.

It’s not difficult to prove that $\phi_{\;n}$ would have a uniform numerical structure independent from which of the 3rd and higher order equation is chosen. On the other hand, lower order equations are obviously a special case of 3rd order equation. Therefore, the conclusion based on 3rd equation is suitable for the model based on the different order of equations too.

As the computation of Equation (22) is centered on the 18 × 18 matrix $A_{n}=A\left(t_{n}\right)$, it is necessary to inspect the numerical characteristics of $A_{n}$ before computational optimization: matrix $A_{n}$, in which only 64 of the total 324 elements are non-zero, is clearly a sparse matrix. A substantial reduction of useless multiplying-zero operations can be achieved by directly expanding $A_{n}$ and operating every element in deduction (namely “direct derivation”), but because of the high order, direct derivation is quite complicated to handle and is not advisable in practice. Fortunately, the 3-order-block form of $A_{n}$, having 23 zero blocks among the total of 36 blocks (see Equation (4)), is still a sparse matrix structurally. Therefore, we can carry out our offline optimization using an indirect method, in which the blocks instead of the elements are treated as the atomic unit in deduction. In this way, we can largely decrease the derivation complexity and retain most of the efficiency of a direct method at the same time. For these reasons, the indirect method is applied to all the derivation processes in this paper. In addition, the nonzero blocks $A_{i3}$ and $A_{IMUii}$ (see Equation (6)), which need few operations in multiplication by any 3-order block (no more than 3^2^ float-pointing multiplications and 2 × 3 additions), are also introduced to the derivation for higher efficiency.

It is a fact that manual derivation with indirect method is still a cumbersome job in view of multiple matrix multiplications (e.g., $A_{n}^{2}$, $A_{n}^{3}$), so we handle our derivation by a computer program instead. With the powerful symbolic-computation function of some well-known math software, e.g., MATLAB and Maple, the complicated derivation can be easily accomplished. After necessary programming, the final derivation results are shown in Equations (23) and (24):

(23) $$A_{n}^{2}={\left[\begin{array}{cccccc} S_{11} & S_{12} & S_{13} & S_{14} & S_{15} & S_{16}\\ S_{21} & S_{22} & S_{23} & S_{24} & S_{25} & S_{26}\\ S_{31} & S_{32} & S_{33} & O_{3\times 1} & O_{3\times 3} & S_{36}\\ O_{3\times 3} & O_{3\times 3} & O_{3\times 3} & O_{3\times 3} & O_{3\times 3} & O_{3\times 3}\\ O_{3\times 3} & O_{3\times 3} & O_{3\times 3} & O_{3\times 3} & S_{55} & O_{3\times 3}\\ O_{3\times 3} & O_{3\times 3} & O_{3\times 3} & O_{3\times 3} & O_{3\times 3} & S_{66} \end{array}\right]}_{18\times 18}$$

(24) $$A_{n}^{3}={\left[\begin{array}{cccccc} T_{11} & T_{12} & T_{13} & T_{14} & T_{15} & T_{16}\\ T_{21} & T_{22} & T_{23} & T_{24} & T_{25} & T_{26}\\ T_{31} & T_{32} & T_{33} & T_{34} & T_{35} & T_{36}\\ O_{3\times 3} & O_{3\times 3} & O_{3\times 3} & O_{3\times 3} & O_{3\times 3} & O_{3\times 3}\\ O_{3\times 3} & O_{3\times 3} & O_{3\times 3} & O_{3\times 3} & T_{55} & O_{3\times 3}\\ O_{3\times 3} & O_{3\times 3} & O_{3\times 3} & O_{3\times 3} & O_{3\times 3} & T_{66} \end{array}\right]}_{18\times 18}$$

Also, the computation of blocks $S_{ij}$ and $T_{ij}$ is described in a computerized sequence as follows:

(25) $$\begin{array}{l} S_{ij}=\sum_{k=1}^{3}A_{ik}A_{kj},\;\;i,j\text{ = }1,2,3\\ S_{ij}=A_{i(j/2-1)}C_{B}^{N},\;\;i\text{ = }1,2,3;\;\text{j = }4,6\\ S_{i5}=S_{i4},\;\;i,j=1,2\\ S_{15}=S_{15}+\;{\left(C_{B}^{N}A_{IMU22}\right)}_{Sim}\\ S_{26}=S_{26}+\;{\left(C_{B}^{N}A_{IMU33}\right)}_{Sim}\\ S_{55}={\left(A_{IMU22}^{2}\right)}_{Sim}\\ S_{66}={\left(A_{IMU33}^{2}\right)}_{Sim}\\ T_{ij}=\sum_{k=1}^{3}A_{ik}S_{kj},\;\;i=1,2,3;\;j=1,2,\ldots ,6\\ T_{26}=T_{26}+\;{\left(C_{B}^{N}A_{IMU33}^{2}\right)}_{Sim}\\ T_{55}={\left(A_{IMU22}S_{55}\right)}_{Sim}\\ T_{66}={\left(A_{IMU33}S_{66}\right)}_{Sim} \end{array}$$

whereas all products involving block $A_{31}$, $S_{34}$ or $S_{35}$ are zero blocks $O_{3\times 3}$ and need no real-time computations; products involving the special blocks, e.g., $A_{i3}$, $S_{i3}$ ( single-nonzero –vector block), $A_{IMUii}$, $S_{55}$, or $S_{66}$ (diagonal blocks), are simplified and are partly indicated in symbol ${\left(\right)}_{Sim}$.

Substituting of $A_{n}$ Equation (4), $A_{n}^{2}$ Equation (23), and $A_{n}^{3}$ Equation (24) in $\phi_{\;n}$ Equation (22), yields:

(26) $$\phi_{\;n}={\left[\begin{array}{cccccc} \phi_{\;11} & \phi_{\;12} & \phi_{\;13} & \phi_{\;14} & \phi_{\;15} & \phi_{\;16}\\ \phi_{\;21} & \phi_{\;22} & \phi_{\;23} & \phi_{\;24} & \phi_{\;25} & \phi_{\;26}\\ \phi_{\;31} & \phi_{\;32} & \phi_{\;33} & \phi_{\;34} & \phi_{\;35} & \phi_{\;36}\\ O_{3\times 3} & O_{3\times 3} & O_{3\times 3} & I_{3\times 3} & O_{3\times 3} & O_{3\times 3}\\ O_{3\times 3} & O_{3\times 3} & O_{3\times 3} & O_{3\times 3} & \phi_{\;55} & O_{3\times 3}\\ O_{3\times 3} & O_{3\times 3} & O_{3\times 3} & O_{3\times 3} & O_{3\times 3} & \phi_{\;66} \end{array}\right]}_{18\times 18}$$

whereas:

(27) $$\begin{array}{l} \phi_{\;ij}=T_{n}A_{ij}+\frac{T_{n}^{2}}{2!}S_{ij}+\frac{T_{n}^{3}}{3!}T_{ij},\;i\in \left[1,3\right],j\in \left[1,6\right],i\neq j\\ \phi_{\;ii}=I_{3\times 3}+T_{n}A_{ii}+\frac{T_{n}^{2}}{2!}S_{ii}+\frac{T_{n}^{3}}{3!}T_{ii},\;\;\;i=1,2,3,5,6 \end{array}$$

Here, $\phi_{\;13}$ and $\phi_{\;23}$ are single-nonzero-column blocks; $\phi_{\;33}$ is the sum of $I_{3\times 3}$ and single-nonzero-column block; $\phi_{\;55}$ and $\phi_{\;66}$ are diagonal blocks. These blocks would be specially used in other processes.

In conclusion, with offline derivation based on 3rd order block method, nearly half amount of blocks $\phi_{\;ij}$ is proved to be constant blocks: $O_{3\times 3}$ or $I_{3\times 3}$ (see Equation (26)), thus, the computations on them are simply eliminated. Additionally, by using the properties of special blocks $A_{i3}$, $S_{i3}$, $T_{i3}$, $A_{jj}$, $S_{jj}$, $T_{jj}$ and introducing the intermediate variables $A_{n}^{2}$, $A_{n}^{3}$, real-time computations on the other half can be further reduced.

### 3.2. Compute $\Gamma_{n}Q_{n}\Gamma_{n}^{T}$

According to [16,17,18,19,20,21,22], the 2nd order approximate of $\Gamma_{n}Q_{n}\Gamma_{n}^{T}$ are computed as follows:

(28) $$\Gamma_{n}Q_{n}\Gamma_{n}^{T}=\frac{T_{n}}{2}\left(Q_{1n}+\phi_{\;n}Q_{1n}\phi_{\;n}^{T}\right)$$

where:

(29) $$Q_{1n}=G\left(t\right)Q\left(t\right)G{\left(t\right)}^{T}={\left[\begin{array}{cccc} C_{B}^{N}Q_{11}{\left(C_{B}^{N}\right)}^{T} & O_{3\times 9} & O_{3\times 3} & O_{3\times 3}\\ O_{9\times 3} & O_{9\times 9} & O_{9\times 3} & O_{9\times 3}\\ O_{3\times 3} & O_{3\times 9} & Q_{22} & O_{3\times 3}\\ O_{3\times 3} & O_{3\times 9} & O_{3\times 3} & Q_{33} \end{array}\right]}_{18\times 18}$$

Obviously, $Q_{1n}$ and $\Gamma_{n}Q_{n}\Gamma_{n}^{T}$ are both symmetric. Thus, for matrix $\Gamma_{n}Q_{n}\Gamma_{n}^{T}$, only its upper (or lower) triangle elements need to be computed in real time. Substitution of $Q_{1n}$ (Equation (29)) in $\Gamma_{n}Q_{n}\Gamma_{n}^{T}$ (Equation (28)), yields:

(30) $$\Gamma_{n}Q_{n}\Gamma_{n}^{T}=\frac{T_{n}}{2}{\left[\begin{array}{cccccc} Q_{n11} & Q_{n12} & Q_{n13} & O_{3\times 3} & Q_{n15} & Q_{n16}\\ Q_{n12}^{T} & Q_{n22} & Q_{n23} & O_{3\times 3} & Q_{n25} & Q_{n26}\\ Q_{n13}^{T} & Q_{n23}^{T} & Q_{n33} & O_{3\times 3} & Q_{n35} & Q_{n36}\\ O_{3\times 3} & O_{3\times 3} & O_{3\times 3} & O_{3\times 3} & O_{3\times 3} & O_{3\times 3}\\ Q_{n15}^{T} & Q_{n25}^{T} & Q_{n35}^{T} & O_{3\times 3} & Q_{n55} & O_{3\times 3}\\ Q_{n16}^{T} & Q_{n26}^{T} & Q_{n36}^{T} & O_{3\times 3} & O_{3\times 3} & Q_{n66} \end{array}\right]}_{18\times 18}$$

where:

(31) $$\begin{array}{l} Q_{nij}=\frac{T_{n}}{2}\left[\phi_{\;i1}Q_{11}^{\prime }\phi_{\;j1}^{T}+\phi_{\;i5}{\left(Q_{22}\phi_{\;j5}^{T}\right)}_{Sim}+\phi_{\;i6}{\left(Q_{33}\phi_{\;j6}^{T}\right)}_{Sim}\right],\;i,j=1,2,3;i\leq j\\ Q_{n11}=Q_{n11}+\frac{T_{n}}{2}Q_{11}^{\prime }\\ Q_{nij}=\frac{T_{n}}{2}{\left(\phi_{\;ij}Q_{j-3,j-3}\phi_{\;jj}\right)}_{Sim},\;i=1,2,3;j=5,6\\ Q_{n55}=\frac{T_{n}}{2}{\left(Q_{22}+\phi_{\;55}Q_{22}\phi_{\;55}\right)}_{Sim}\\ Q_{n66}=\frac{T_{n}}{2}{\left(Q_{33}+\phi_{\;66}Q_{33}\phi_{\;66}\right)}_{Sim} \end{array}$$

In Equation (31), $Q_{n55}$ and $Q_{n66}$ are apparently diagonal blocks that may be used to simplify other processes. As stated above, we optimize the computation of $\Gamma_{n}Q_{n}\Gamma_{n}^{T}$ mainly by applying some special numerical properties to the offline derivation (e.g., the sparsity of G(t), the simplicity of diagonal blocks $Q_{22}$, $Q_{33}$, $\phi_{\;55}$, $\phi_{\;66}$, and the symmetry of $\Gamma_{n}Q_{n}\Gamma_{n}^{T}$) and much of the computation time is saved.

### 3.3. Compute $P_{n}\left(-\right)$

For simplicity, $P_{n}\left(-\right)$ and $P_{n}\left({+}_{e}\right)$ will be written in a unified form $P_{n}$ (this is allowed because of that $P_{n}\left(-\right)$ and $P_{n}\left({+}_{e}\right)$ are actually the same covariance matrices in different state and share the same memories). Similar to $\phi_{\;n}$ or $\Gamma_{n}Q_{n}\Gamma_{n}^{T}$, $P_{n}$ is written in 3rd-order-block-matrix form:

(32) $$P_{n}={\left[\begin{array}{cccc} P_{n,11} & P_{n,12} & \cdots & P_{n,16}\\ P_{n,21} & P_{n,22} & \cdots & P_{n,26}\\ \vdots & \vdots & \ddots & \vdots \\ P_{n,61} & P_{n,62} & \cdots & P_{n,66} \end{array}\right]}_{18\times 18}$$

According to its definition, $P_{n}$ is a symmetric matrix:

(33) $$P_{n,ij}=P_{n,ji}^{T}\;\;\;,\;\;\;i,j=1,2\ldots 6$$

Thus, only the lower (or upper) triangle part needs substantive computation. Regardless of $\Gamma_{n}Q_{n}\Gamma_{n}^{T}$ at first, Equation (19) is written as:

(34) $$P_{n}\left(-\right)=\phi_{\;n}P_{n-1}\left({+}_{e}\right)\phi_{\;n}^{T}$$

Substitution of Equation (26) and (32), yields:

(35) $$\begin{array}{l} P_{n,11}=\sum_{i=1}^{6}\left\{\phi_{\;1i}{\left[\sum_{j=1}^{6}\left(P_{n-1,ij}\phi_{\;1j}^{T}\right)\right]}_{Temp}\right\}\\ P_{n,21}=\sum_{i=1}^{6}\left\{\phi_{\;2i}{\left[\sum_{j=1}^{6}\left(P_{n-1,ij}\phi_{\;1j}^{T}\right)\right]}_{Temp}\right\}\\ \vdots \\ P_{n,61}={\left[\phi_{\;66}\sum_{j=1}^{6}{\left(P_{n-1,6j}\phi_{\;1j}^{T}\right)}_{Rep}\right]}_{Sim}\\ P_{n,22}=\sum_{i=1}^{6}\left\{\phi_{\;2i}{\left[\sum_{j=1}^{6}\left(P_{n-1,ij}\phi_{\;2j}^{T}\right)\right]}_{Temp}\right\}\\ P_{n,32}=\sum_{i=1}^{6}\left\{\phi_{\;3i}{\left[\sum_{j=1}^{6}\left(P_{n-1,ij}\phi_{\;2j}^{T}\right)\right]}_{Temp}\right\}\\ \vdots \\ P_{n,62}={\left[\phi_{\;66}\sum_{j=1}^{6}{\left(P_{n-1,6j}\phi_{\;2j}^{T}\right)}_{Rep}\right]}_{Sim}\\ \vdots \\ P_{n,66}={\left(\phi_{\;66}P_{n-1,66}\phi_{\;66}\right)}_{Sim} \end{array}$$

where the symbol ${\left(\right)}_{Temp}$ represents intermediate items that would be temporarily stored in memory to avoid repeated computation; the symbol ${\left(\right)}_{Rep}$ represents the repeated item that have been computed before and are available by directly accessing ${\left(\right)}_{Temp}$ and the symbol ${\left(\right)}_{Sim}$ indicates that the special properties of $\phi_{\;i3}^{T}$ are exploited to perform further optimization.

After Equation (35) is done, $\Gamma_{n}Q_{n}\Gamma_{n}^{T}$ should be added. Substitution of Equations (30) and (32) in Equation (19), yields:

(36) $$P_{n,ij}=P_{n,ij}+Q_{n,ij},\;i,j=1,2,3,5,6;\;i\geq j$$

Above all, the computation of $P_{n}\left(-\right)$ is divided into two steps: Equations (34) and (36). As the latter has few computations, optimization mainly focuses on the former. Employing the sparsity of $\phi_{\;n}$, the symmetry of $P_{n}\left(-\right)$ and the repeatability of intermediate items, the real time of calculating Equation (34) is greatly improved.

### 3.4. Compute $K_{n}$

Substitution of Equations (11), (12) and (32) in the inversion part of Equation (20), yields:

(37) $$H_{n}P_{n}\left(-\right)H_{n}^{T}+R_{n}={\left[\begin{array}{cc} H_{13}P_{n,33}H_{13}+R_{11} & H_{13}P_{n,23}^{T}\\ P_{n,23}H_{13} & P_{n,22}+R_{22} \end{array}\right]}_{6\times 6}$$

Since $H_{n}P_{n}\left(-\right)H_{n}^{T}+R_{n}$ has a low order, few computations are needed during inversion (the complexity is $O(6^{3})$ that can be neglected in comparison with other procedures) and the details of inversion are not discussed accordingly. Here, we use the symbol D to represent the result of inversion directly:

(38) $$D={\left[H_{n}P_{n}\left(-\right)H_{n}^{T}+R_{n}\right]}^{-1}$$

Matrix D is easily proved to be symmetric and can be written in the 3-order-block-matrix form as:

(39) $$D={\left[\begin{array}{cc} D_{11} & D_{12}\\ D_{12}^{T} & D_{22} \end{array}\right]}_{6\times 6}$$

Then $K_{n}$ can be computed as:

(40) $$K_{n}={\left[\begin{array}{cc} P_{n,12}D_{12}^{T}+P_{n,13}H_{13}D_{11} & P_{n,12}D_{22}+P_{n,13}H_{13}D_{12}\\ P_{n,22}D_{12}^{T}+P_{n,23}H_{13}D_{11} & P_{n,22}D_{22}+P_{n,23}H_{13}D_{12}\\ \vdots & \vdots \\ P_{n,62}D_{12}^{T}+P_{n,63}H_{13}D_{11} & P_{n,62}D_{22}+P_{n,63}H_{13}D_{12} \end{array}\right]}_{18\times 6}$$

Define

$$K_{n}$$

as:

(41) $$K_{n}={\left[\begin{array}{cc} K_{11} & K_{12}\\ K_{21} & K_{22}\\ \vdots & \vdots \\ K_{61} & K_{62} \end{array}\right]}_{18\times 6}$$

then:

(42) $$K_{ij}=P_{n,i2}D_{j2}^{T}+P_{n,i3}H_{13}D_{1j},\;i=1,2,\ldots ,6;\;j=1,2$$

The derivation result shows that, by using the block-matrix optimization technique, only a simple process (Equation (37), (38) and (42)) is needed in $K_{n}$ updating. Compared with the complicated Equation (20) which needs multiple 18-order matrix production, our method seems much more simple and efficient in computing.

### 3.5. Compute $P_{n}\left({+}_{e}\right)$

Transform Equation (21) into:

(43) $$P_{n}\left({+}_{e}\right)=P_{n}\left(-\right)-K_{n}H_{n}P_{n}\left(-\right)$$

Obviously, the pressure in computation mainly comes from $K_{n}H_{n}P_{n}\left(-\right)$. Fortunately, $K_{n}H_{n}P_{n}\left(-\right)$ is a symmetrical matrix (this is an evident inference according to Equation (43) in the condition that $P_{n}\left({+}_{e}\right)$, $P_{n}\left(-\right)$ are both symmetrical) so that only its upper (or lower) triangular part needs real-time updating. Define $K_{n}H_{n}P_{n}\left(-\right)$ as the variance’s increment $\Delta P_{n}$:

(44) $$\Delta P_{n}=K_{n}H_{n}P_{n}\left(-\right)={\left[\begin{array}{cccc} \Delta P_{11} & \Delta P_{12} & \cdots & \Delta P_{16}\\ \Delta P_{21} & \Delta P_{22} & \cdots & \Delta P_{26}\\ \vdots & \vdots & \ddots & \vdots \\ \Delta P_{61} & \Delta P_{62} & \cdots & \Delta P_{66} \end{array}\right]}_{18\times 18}$$

Substitution of Equations (12), (32) and (41), yields:

(45) $$\Delta P_{ij}=K_{i1}H_{13}P_{n,3j}+K_{i2}P_{n,2j}\;,i,j=1,2,\ldots ,6;i\leq j$$

then:

(46) $$P_{n,ij}=P_{n,ij}-\Delta P_{ij}\;,i,j=1,2,\ldots ,6;i\leq j$$

In conclusion, taking advantage of the symmetry of $P_{n}\left({+}_{e}\right)$ and the sparsity of $H_{n}$, we deduct a simple form of computing $P_{n}\left({+}_{e}\right)$: Equations (45) and (46). Equation (46) is apparently costless as only the matrix subtraction is involved. Equation (45) involves matrix products, but requires no computations on most blocks of $P_{n}$ (except the blocks in 2nd and 3rd rows) and proves to be highly efficient.

### 3.6. Parallel Computation

The optimization results (see Table 2, Table 3, Table 4, Table 5 and Table 6) reveal that, computation on $P_{n}\left(-\right)$ costs nearly half of the processing time and becomes a bottleneck for further efficiency. The low efficiency mainly arises from that $P_{n}\left(-\right)$ becomes increasingly dense and can provide few zero blocks after initial filtering cycles. In low-accuracy applications, some researchers may force the some matrix elements to be zero for low computational costs with the engineering approximate method. However, to some accuracy-demanded applications, methods with more accuracy and efficiency are still looked for. Parallel computation is one of the feasible approaches.

Before discussing the parallel method, we perform an inspection of the whole computation processes in order to clarify the dependency of every parameter on $P_{n}\left(-\right)$. The numerical relationship between $P_{n}\left(-\right)$ and other parameters can be consulted in Table 1.

Except for $P_{n}\left({+}_{e}\right)$, all parameters are either irrelevant to $P_{n,ij}$ or only relevant to 2nd and 3rd block rows (columns) of $P_{n}\left(-\right)$. Taking this fact into account, we can call 2nd and 3rd block rows (columns) “useful” data and treat the rest as “useless” (it is emphasized here that “useless” only refers to the requirement of most computation processes, but not that the data is really useless and needs no updates).

Furthermore, because $P_{n}\left(-\right)$ is a symmetrical matrix, the “useful” blocks are only 2nd, 3rd block columns in fact. If “ × ” represents “useless” blocks, $P_{n}\left(-\right)$ can be written as:

(47) $$P_{n}={\left[\begin{array}{cccccc} \times & P_{n,12} & P_{n,31} & \times & \times & \times \\ \times & P_{n,22} & P_{n,32} & \times & \times & \times \\ \times & P_{n,32} & P_{n,33} & \times & \times & \times \\ \times & P_{n,42} & P_{n,34} & \times & \times & \times \\ \times & P_{n,52} & P_{n,35} & \times & \times & \times \\ \times & P_{n,62} & P_{n,36} & \times & \times & \times \end{array}\right]}_{18\times 18}$$

**Table 1:** Dependency of every real-time parameter on $P_{n}\left(-\right)$ blocks.

$\phi_{\;n}$, $Q_{n}$, $P_{n}\left(-\right)$	$K_{n}$ (Equations (40) and (41))	$P_{n}\left({+}_{e}\right)$ (Equation (43))	$\Delta P_{ij}$ (Equation (45))
Irrelevant to $P_{n,ij}$	Dependent on $P_{n,i2}$, $P_{n,i3}$ only	Dependent on $P_{n,ij}$	Dependent on $P_{n,2i}$, $P_{n,3i}$ only

In measurement updating (Equations (18), (20) and (21)), all parameters except $P_{n}\left({+}_{e}\right)$ are decoupled with the “useless” data (Equation (19)) which belong to the time propagation. Moreover, to the coupled $P_{n}\left({+}_{e}\right)$ its time-consuming part $\Delta P_{ij}$ is also independent of the “useless” blocks. In other words, with some appropriate modification of measurement updating and time propagation, we can produce a new decoupling mechanism of the two procedures. The modification contains three steps:

Step1. Subdivide time propagation.
Classify the blocks of $P_{n}\left(-\right)$ into two kinds: “useful” data and “useless” data;
Step 2. Subdivide measurement updating.
Subdivide $P_{n}\left({+}_{e}\right)$ into two processes: computing $\Delta P_{ij}$ (Equation (45)) and adding $P_{n}\left(-\right)$ (Equation (46));
Step 3. Restructure measurement updating.
Restructure measurement updating process as $\Delta P_{ij}$ (instead of $P_{n}\left({+}_{e}\right)$) (Equations (18) and (21)).

With the above steps, the restructured process and the “useless” data updating process are completely numerically decoupled and can be computed in parallel. In this way, the efficiency bottleneck in $P_{n}\left(-\right)$ updating is broken.

**Figure 2:**
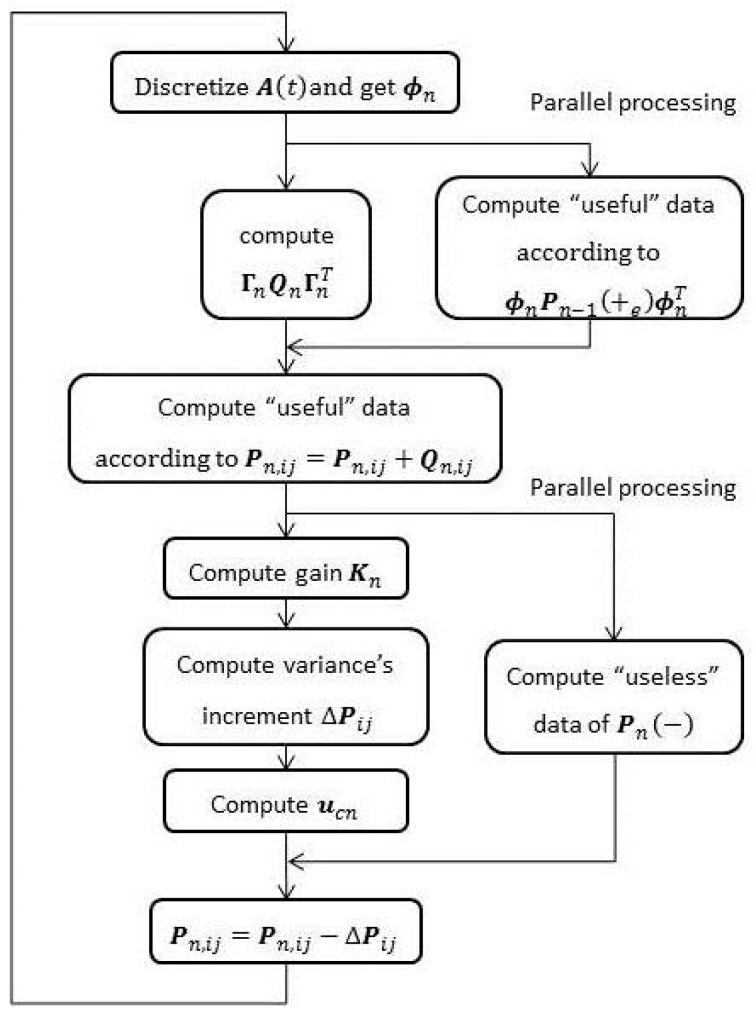
Filtering process with parallel computation.

Furthermore, in order to achieve the parallel computation on the “useful” data as well, we similarly subdivide the process of updating “useful” data into two steps: computing $\phi_{\;n}P_{n-1}\left({+}_{e}\right)\phi_{\;n}^{T}$ (Equation (34)) and adding $\Gamma_{n}Q_{n}\Gamma_{n}^{T}$ (Equation (36)). Apparently, computing $\phi_{\;n}P_{n-1}\left({+}_{e}\right)\phi_{\;n}^{T}$ and updating $\Gamma_{n}Q_{n}\Gamma_{n}^{T}$ (Equation (31), which belongs to time propagation) can be processed in parallel. In summary, the process of Kalman filtering with parallel computation is described as Figure 2.

It is pointed out here that the parallel method in this paper has a fundamental difference with some other parallel methods [23,24]. The others owe their decoupling to the so-called “mandatory delay”, which may cause certain accuracy damage. On the contrary, the decoupling in this paper needs neither “mandatory delay” nor engineering approximation and is achieved mainly by introducing the “useful” data and subdividing the computation process. Essentially, it is the numerical characteristic of SINS/GPS that brings up this special decoupling. Therefore, the parallel processing in this paper is an accuracy-lossless method.

In respect of efficiency, the parallel optimization method can reduce much execution time needed in updating both $\Gamma_{n}Q_{n}\Gamma_{n}^{T}$ and the “useless” blocks (including 918 multiplications and 603 additions in updating $\Gamma_{n}Q_{n}\Gamma_{n}^{T}$, and 1089 multiplications and 1107 additions in updating “useless” blocks). In comparison with non-parallel methods, efficiency of parallel method increases by about 25%.

## 4. Optimization Efficiency

According to the derivation result, we can figure out the optimization efficiency by counting and comparing the computational counts needed in both the proposed method and the existing algorithm. In detail, the discussion about counts proceeds on each parameter updating procedure, respectively. As matrix multiplication is mainly involved, the calculation will focus on the floating-point operations of multiplication and addition. Table 2, Table 3, Table 4, Table 5 and Table 6 will show the statistical details of each parameter. Then, Table 7 and Table 8 give the global efficiency and the comparison with other general algorithms, respectively. As shown in the tables, the offline-derivation and parallel method saves about 90% of computational time and 66% of memory space, while keeping the required accuracy level. Compared with general decomposition optimization algorithms, the proposed method needs no complicated matrix theory but producing higher efficiency. Under the condition that high hardware precision has guaranteed the numerical robustness, our algorithm appears quite simple and practical.

**Table 2:** Optimization efficiency of $\phi_{\;n}$ updating process.

	Offline-Derivation Method	Traditional Method
Multiplication	$42\text{ × }3^{3}\text{ + }36\times 3^{2}\text{ + }13\text{ × }3\text{ × }3^{2}=1809$ (I), 10%	$3\text{ × }18^{3}\text{ + }3\times 18^{2}\text{ = }18468$ (II)
Addition	$42\text{ × }2\text{ × }3^{2}+9\text{ × }2\text{ × }3+13\text{ × }2\text{ × }3^{2}=1044$ (I), 6%	$3\text{ × }17\text{ × }18^{2}\text{ + }3\text{ × }18^{2}\text{ = }17496$ (II)
Memory	$32\text{ × }3^{2}\text{ × }8\text{ Byte}=2.3\text{ KB}$ (III)	$3\text{ × }18^{2}\text{ × }8\;\mathrm{Byte}\;=\text{7.6 KB}$ (III)

In (I), 42 × 3^3^ (42 × 2 × 3^2^), 36 × 3^2^ (9 × 2 × 3) and 13 × 3 × 3^2^ (13 × 2 × 3^2^) represent the counts on multiplication (addition) of items in Equation (25) without special blocks, of items in Equation (25) with special block and of items in Equation (27) respectively, and only 3^2^ & 3^3^ items are counted; In (II), the first item represents the counts on operations of 18-order-matrix multiplication, the second item represents that of multiplication by scalars or matrix addition; In (III), data are assumed to be double-precision (8-byte word length); the percentages represent the efficiency in comparison with the traditional method.

**Table 3:** Optimization efficiency of $\Gamma_{n}Q_{n}\Gamma_{n}^{T}$ updating process.

	Offline-Derivation Method	Traditional Method
Multiplication	$24\times 3^{3}+18\times 3^{2}+12\times 3^{2}=918$, 6%	$2\times 18^{3}+18^{2}+18\times 6^{2}+6\times 18^{2}=14580$
Addition	$24\times 2\times 3^{2}+9\times 2\times 3+13\times 3^{2}=603$, 4%	$2\times 17\times 18^{2}+18^{2}+5\times 6\times 18+17\times 18\times 6=13716$
Memory	$13\times 3^{2}\times 8\text{ Byte}=0.9\text{ KB}$	$3\;\times \;18^{2}\text{ × }8\text{ Byte}=7.6\text{ KB}$

**Table 4:** Optimization efficiency of $P_{n}\left(-\right)$ updating process.

	Offline-Derivation Method	Traditional Method
Multiplication	$120\times 3^{3}+37\times 3^{2}=3573$, 31%	$2\times 18^{3}=11664$
Addition	$120\times 2\times 3^{2}+18\times 2\times 3+134\times 3^{2}=3474$, 31%	$2\times 17\times 18^{2}+18^{2}=11340$
Memory	$60\times 3^{2}\times 8\text{ Byte}=4.2\;\text{KB}$	$3\times 18^{2}\times 8\;\text{Byte}=7.6\;\text{KB}$

**Table 5:** Optimization efficiency of $K_{n}$ updating process.

	Offline-Derivation Method	Traditional Method
Multiplication	$24\times 3^{3}+6\times 3^{2}=702$, 11%	18 × 18 × 6 + 18 × 6 × 6 + 6 × 18 × 18 + 6 × 18 × 6 = 5184
Addition	$24\times 2\times 3^{2}+12\times 3^{2}=540$, 11%	17 × 18 × 6 + 17 × 6 × 6 + 5 × 18 × 6 + 17 × 18 × 6=4824
Memory	$16\;\times \;3^{2}\;\times \;8\;\text{Byte}=\;1.1\;\text{KB}$	（3 × 6 × 18+6 × 6）× 8 Byte = 2.8 KB

**Table 6:** Optimization efficiency of $P_{n}\left({+}_{e}\right)$ updating process.

	Offline-Derivation Method	Traditional Method
Multiplication	$42\times 3^{3}+6\times 3^{2}=1188$, 15%	$6\times 18\times 18+18^{3}=7776$
Addition	$42\times 2\times 3^{2}+42\times 3^{2}=1134$, 15%	$5\times 18^{2}+17\times 18^{2}+18^{2}=7452$
Memory	$16\times 3^{2}\times 8\;\text{Byte}=1.1\;\text{KB}$	（3×6×18+6×6）×8 Byte=2.8 KB

**Table 7:** Optimization efficiency of offline-derivation and parallel method.

	Offline-Derivation & Parallel Method	Traditional Method
Multiplication	1809+2484+702+1188=6183 (IV), 10.7%	18468+14580+11664+5184+7776=57672
Addition	1044+2475+540+1134=5193 (IV), 9.5%	17496+13716+11340+4824+7452=54828
Memory	2.3+0.9+4.2+1.1+1.1=9.6 KB	7.6+7.6+7.6+2.8+2.8=28.4 KB

In (IV), 2484 (2475) is the operation times of “useful” blocks updating.

**Table 8:** Comparison of offline-derivation & parallel method and general optimization filters.

	Multiplication	Addition
SRIF filtering	$\frac{7}{6}\times 18^{3}+36\times 18^{2}=18468$ (V)	$\frac{7}{6}\times 18^{3}+36\times 18^{2}=18468$ (V)
U-D decomposing filtering	$\frac{1}{2}\times 18^{3}+\frac{1}{2}\times 18^{2}+10\times 18^{2}+8\times 18=6462$ (VI)	$\frac{1}{2}\times 18^{3}+\frac{1}{2}\times 18^{2}+9\times 18^{2}+9\times 18=6156$ (VI)
SVD filtering	$26\times 18^{3}+78\times 18^{2}=176904$ (VII)	$26\times 18^{3}+78\times 18^{2}=176904$ (VII)
offline-derivation and parallel method	2484+702+1188=4374 (VIII)	2475+540+1134=4149 (VIII)

(V) is explained in Reference [3], while (VI) in Reference [25], Reference [26] and (VII) in Reference [5]; Point out here that, (VI) relies on the precondition that $\phi_{\;n}$ is already an upper-triangle matrix in 9-order-block form (what is the conclusion of offline derivation), otherwise, 9702 multiplications and 9396 additions are actually needed; concerning (VIII), the procedure of computing $\phi_{\;n}$ is no longer counted as no optimizations are against $\phi_{\;n}$ in the general algorithms.

## 5. Simulation

To evaluate the method validity, we design a practical loosely-coupled SINS/GPS program in CCS v5.5 (a well-known programming tool on DSP platform). The program comprises three modules:

(1) Sensor data sampling module.
(2) SINS algorithm module. This module is designed to implement the calculation of attitude, velocity and position independently (only the inertial sensors data is needed).
(3) Kalman filter module. This part is for data fusion. The system module is designed according to Section 2, and the computation process is programmed using the offline and parallel method described in Section 3. Besides, for accuracy evaluation, the classical KF is also designed as a control group.

With the developed program, we can simulate filtering process and evaluate the performance of our method in CCS. The simulation parameters and conditions are set as follows:

(1) DSP TMS320C6722, a famous float-point CPU, is chosen in CCS as the computing device.
(2) The carrier of the navigation system is assumed to be static. With this assumption, the gyro/accelerometer would detect no valid rotate/velocity rate aside from devices noise. Therefore, noise is considered the sampling data driving the SINS/GPS program. As the carrier is static, velocity/position should stay at the zero/initial value in theory, thus, this assumption can largely facilitate the evaluation of program results.
(3) Velocity/position measured by GPS is constantly set:
  $$v_{GPS}\equiv {\left(0,0,0\right)}^{T},\;\left(L\;\lambda \;h\;\right){\;}_{GPS}^{T}\equiv {\left(\begin{array}{ccc} 0.6977686 & 2.0306152 & 90.0 \end{array}\right)}^{T}$$
  Velocity/position calculated by SINS is initialized:
  $$v_{SINS}={\left(0,0,0\right)}^{T},\;\left(L\;\lambda \;h\;\right){\;}_{SINS}^{T}={\left(\begin{array}{ccc} 0.6977688370723981 & 2.030615370778482 & 91.0 \end{array}\right)}^{T}$$
(4) IMU data rate (noise frequency): 100 Hz; SINS velocity/position updating rate: 50/20 Hz; filtering rate: 10 Hz.

**Figure 3:**
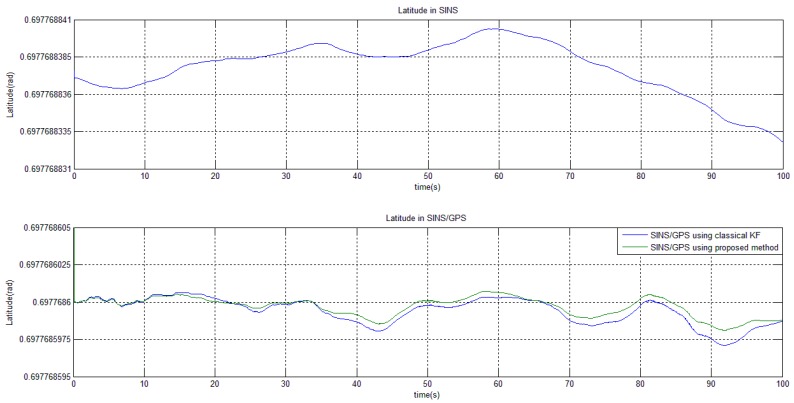
Latitude calculation by different methods.

**Figure 4:**
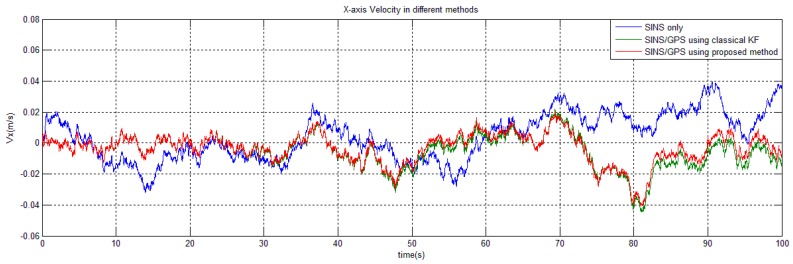
Velocity calculation by different methods.

With the above setting, we run the program in CCS for 100 s and record the navigation outputs (e.g., the X-axis velocity and latitude). The simulation results are illustrated in Figure 3 and Figure 4.

The results show that the proposed method effectively restrains the unbounded error accumulation in SINS and keep the estimated variables around their mean values. On the aspect of estimation performance, our method is nearly at par to the classical KF (the proposed-method curve does not completely overlap the classical-KF one because of less calculation and less truncation errors). This conclusion agrees with the theoretical analysis: our numerical method, needing no modification of the system module and no engineering approximation, causes little damage to the estimation accuracy, but from the point of view of real-time computation, our method is much more efficient than the classical KF. In the DSP program, the proposed method needs 64,820 system clocks in each filtering cycle while classical KF needs 390,480 clocks. If a clock frequency of 200 MHz, the typical frequency of a DSP TMS320C6722 is chosen, then our method will cost only 324 μs in each filtering operation. Such a level of real-time efficiency is highly valuable to the many accurate navigation applications.

## 6. Conclusions

Starting from a common classical-Kalman-fitler-based SINS/GPS model (position-speed integration with 18 states and six measurements), we present an optimization algorithm based on the system’s numerical characteristics. The algorithm employs a block-matrix technique in offline derivation, where special blocks (e.g., zero blocks, diagonal blocks, *etc.*) are used to simplify the calculation. In this way, plenty of invalid multiplying by zero and repeated operations are avoided offline. Furthermore, aiming at the time-consuming update of $P_{n}\left(-\right)$, a novel parallel method is implemented by defining “useful” data and subdividing computational process, and the entire filtering procedure is greatly accelerated. The statistical analysis and simulation results reveal that the offline algorithm, coupled with the parallel method. can reduce the CPU processing time by 90% and the memory usage by 66%. Compared with several general decomposition-optimization algorithms, the proposed method requires no complex matrix theory rather more efficiency. The distinguished feature of the algorithm is that no modification of the system module and no engineering approximation are needed, thus it causes little harm to the base filter. It is pointed out that although the derivation is based on a specific integration model, the algorithm as a complete numerical optimization approach can be transplanted to other advanced models of SINS/GPS or other extended filters. With the powerful symbolic-operation function of the MATLAB program, researchers can manipulate formulas involving high order matrices in an easy way. Consequently, the proposed method is an engineering-tractable approach with high efficiency and high precision for SINS/GPS integrated navigation systems.
